# Supplementary material for: Energy dissipation on magic angle twisted bilayer graphene
Source: Commun Phys. 2023 Nov 28;6(1):344. doi: 10.1038/s42005-023-01441-4 (PMC11041686; doi:10.1038/s42005-023-01441-4)
Supplement: Supplementary file 2 — Supplementary Material [file 42005_2023_1441_MOESM2_ESM.pdf]

***Supplementary Note*: Energy dissipation on magic angle twisted bilayer graphene**

Alexina Ollier,[1,2,*] Marcin Kisiel,[1,†] Xiaobo Lu,[3,‡] Urs Gysin,[1,§]
Martino Poggio,[1,2,¶] Dmitri K. Efetov,[4,**] and Ernst Meyer[1,††]

[1]*Department of Physics, University of Basel, Klingelbergstrasse 82, CH-4056 Basel, Switzerland*

[2]*Swiss Nanoscience Institute, Klingelbergstrasse 82, CH-4056 Basel, Switzerland*

[3]*International Center for Quantum Materials, Collaborative Innovation Center of Quantum Matter, Peking University, 100871, Beijing, China*

[4]*Department of Physics, Ludwig-Maximilians-University München, Geschwister-Scholl-Platz 1, 80539 München, Germany*

(Dated: October 16, 2023)

SUPPLEMENTARY NOTE 1: DESCRIPTION OF PENDULUM AFM AND DISSIPATION MECHANISMS

Pendulum AFM (p-AFM) is a home build microscope dedicated to the measurement of extremely small forces in order of aN. In the pendulum geometry the cantilever is oscillating perpendicularly to the sample surface, "shearing" the nanometer vacuum gap between tip and the sample. The geometry allows to use very soft cantilevers with spring constants $k = 10^{-5} - 10^{-3}\ Nm^{-1}$ and to avoid snapping into the contact with the sample surface. The force sensitivity is given by:

$$F_{min} = \sqrt{\frac{2k_BTk \cdot BW}{\pi f Q}} \tag{S1}$$

where k_B, T, BW, f, and Q are Boltzmann constant, temperature, measurement bandwidth, cantilever frequency and quality factor, respectively. p-AFM operates at cryogenic temperatures of $T = 5K$ and under ultra high vacuum (UHV) conditions $p = 10^{-10}$ mbar. Very high quality factors equal to about $Q = 10^5 - 10^6$ are achieved after the annealing of the sensors up to 600 $\circ C$ for 12 hours. Long term annealing removes the weakly bounded molecules from the cantilever surface and leads to the reduction of the static charges or trap states present on the surface of the cantilever. High quality factors and low stiffness of the sensors result in very low internal power dissipation inside the cantilever:

$$P_0 = \frac{\pi k A^2}{eQ} \approx \mu eV/cycle \tag{S2}$$

where A and e are the oscillation amplitude and elementary charge, respectively. The dissipated power is few orders of magnitude smaller as compared to stiffer sensors operating in standard AFM geometry. The microscope can be operated in a variable temperature range of 4-300K and external magnetic field (± 7T) can be applied axially to the sensor. In addition to AFM, the microscope is also furnished with the Scanning Tunneling (STM) line and in STM mode a metal coated and stiffer cantilevers must be used.

The motion of the cantilever is controlled by means of the phase lock loop (PLL) electronic feedback circuit, where the frequency caused by the tip sample interaction is measured. PLL essentially creates a replica of the oscillation signal and feed it to the cantilever piezoelectric shaker with a proper phase shift. The amplitude of the oscillator is set to be constant and

it is controlled by amplitude controller feedback circuit which applies certain amount of excitation voltage A_{exc} to the shaker in order to maintain constant oscillation amplitude. Thus, whenever energy loss between tip and sample occurs the driving voltage A_{exc} increases. The energy loss is measured according to the formula:

$$P = P_0 \left(\frac{A_{exc}(d)}{A_{exc,0}} - \frac{f(d)}{f_0} \right) \tag{S3}$$

where $A_{exc}(d)$ and $f(d)$ are distance dependent excitation amplitude and frequency of the oscillating cantilever, while the suffix zero refers to the free cantilever. Details about the p-AFM microscope are given elsewhere [1].

Three main different channels of energy loss are shown in Fig. **S1**. We can distinguish Joule dissipation (a) which involves creation of the local currents when the charged tip oscillates over a resistive medium, van der Waals dissipation (b) which arises from fluctuating electromagnetic fields between tip and sample. The oscillating tip senses different electromagnetic force every oscillating cycle which according to fluctuation-dissipation theorem results in dissipation rise. Phononic dissipation (c) is due to creation of time dependent elastic deformation. The energy is lost to the creation of acoustic, longitudinal phonons when the moving tip drags the deformation across the surface. The Joule dissipation is the

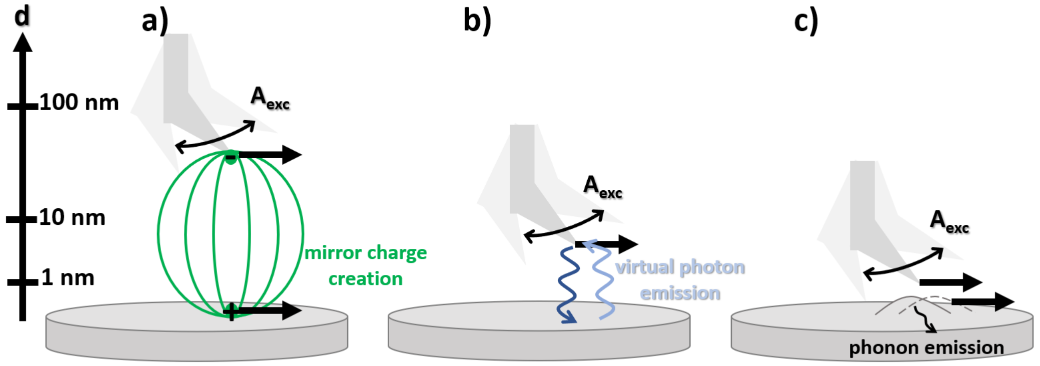

Figure S1. Schematics of the p-AFM oscillating over the surface and the three main dissipation mechanisms. a) is the Joule dissipation mechanism and the energy loss is due to creation of local, displacement currents induced when the charged tip oscillates over a resistive medium, b) shows van der Waals dissipation which is due to the surface charge fluctuations and c) depicts the phononic friction where the surface deformation is dragged by moving the tip and the energy is lost to the creation of longitudinal, acoustic phonons.

dominant dissipation channel at large tip-sample separations, whereas the phononic drag usually occurs at distances of few nanometers [2, 3].

SUPPLEMENTARY NOTE 2: BACKGATE VOLTAGE CONVERSION INTO DOPING CONCENTRATION

The twisted bilayer graphene (tBLG) is deposited on p-doped silicon substrate with resistivity $\rho < 0.005\Omega cm$. The charge concentration was controlled with a DC voltage applied to the silicon backgate, whereas tBLG was coupled capacitively via $d_{SiO} = 300$ nm SiO_2 oxide and $d_{hBN} = 10$ nm of hexagonal boron nitride - hBN. Whereas for positive voltages applied to Si backgate the backgate capacitance is geometric, the application of negative voltages to the p-doped substrate results in a creation of a depletion region that leads to voltage dependent backgate capacitance. Figure **S2** a) shows the capacitance model for a metal-oxide-semiconductor (MOS) system. The accumulation, depletion and inversion regions are visible. In the accumulation phase, the capacitance is constant and determined only by sample geometry and equal to $C_i = \epsilon_i/d$ where ϵ_i is the permittivity of the material and d is the oxide thickness. As the voltage rises the depletion region starts to appear, inducing

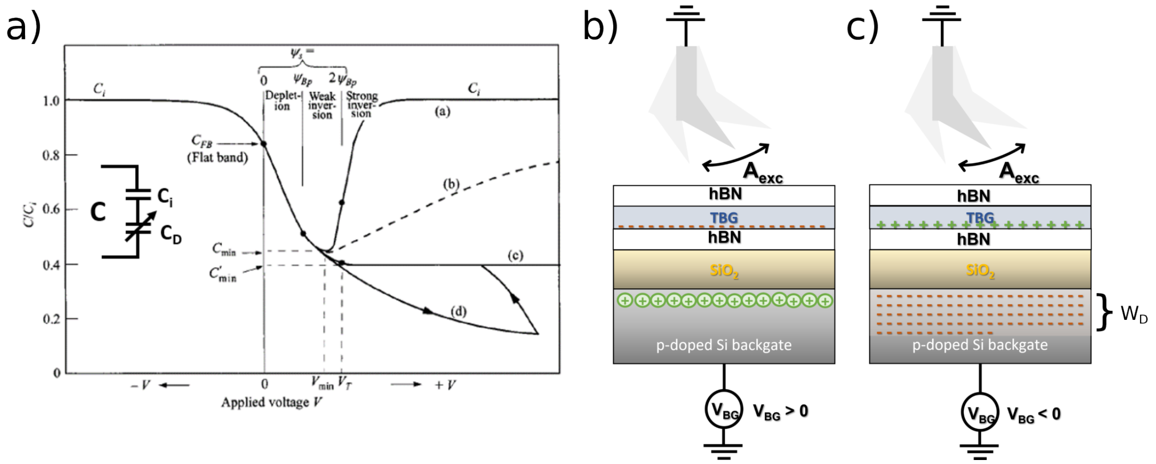

Figure S2. — a) Creation of depletion layer for MOS system. The graph was taken from [4], b) is the schematics showing accumulation regime where the electrons (-) gets accumulated at the sample surface when a positive backgate voltage is applied. c) shows creation of the depletion region, the majority carriers - holes gets repelled from the surface creating the depletion region and let the surface to be p-doped.

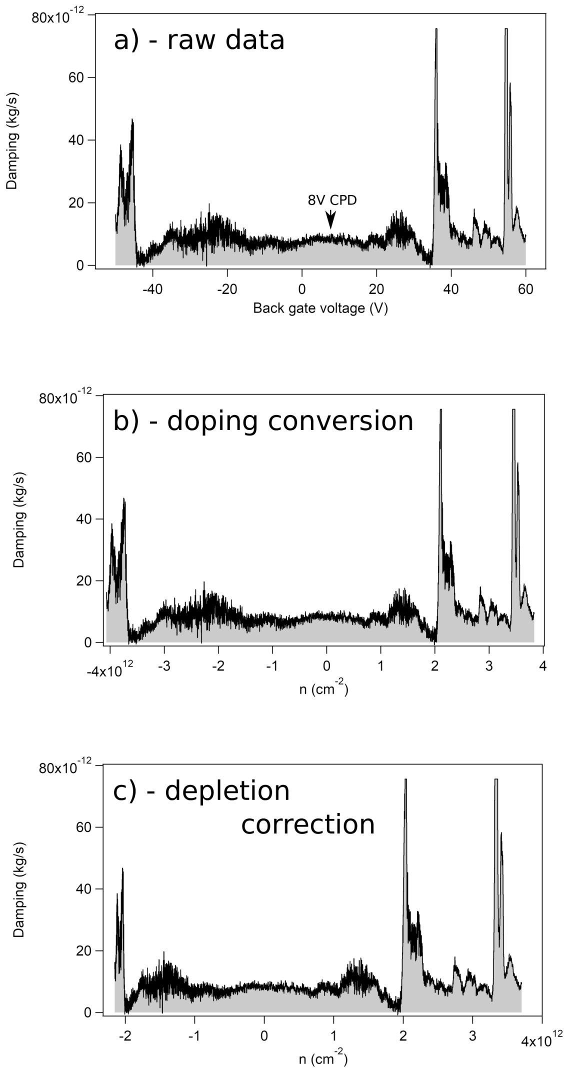

Figure S3. — Raw data dissipation curve before (a) and after (b) depletion layer correction.

the drop of the capacitance value. For p-doped substrate the creation of accumulation and depletion regions is schematically shown in Fig **S2** (b) and (c), respectively. For negative voltage applied to the substrate the holes get repelled from the bilayer graphene, increasing the thickness of the insulating layer. The depletion width in noted as W_D and the depletion capacitance C_D has to be taken into account when calculating the total system capacitance C.

The depletion width (W_D) and capacitance (C_D) were calculated as following [4]:

$$W_D = \sqrt{\frac{\epsilon_s^2}{C_{ox}^2} + \frac{2\epsilon_s V_{BG}}{qN_A}} - \frac{\epsilon_s}{C_{ox}} \tag{S4}$$

$$C_D = \frac{\epsilon_s}{W_D} \tag{S5}$$

where $\epsilon_s = 11.7$ is the silicon permittivity, C_{ox} is the oxide and hBN capacitance, N_A is the acceptor concentration and V_{BG} is the backgate voltage.

The total capacitance in depletion region is calculated according to formula:

$$C = \frac{C_i C_D}{C_i + C_D} \tag{S6}$$

The raw and the corrected dissipation spectra are presented on figure **S3** is (a) and (b) respectively. The depletion correction was used to correct the width and the position of the dissipation peaks in the hole doping part.

SUPPLEMENTARY NOTE 3: FREQUENCY SHIFT PARABOLA

Figure **S4** (a) shows the raw frequency shift data taken simultaneously with the dissipation spectrum. The curve is composed of two distinct parabolas. The parabola has a minimum at $n_D = 0.29 \times 10^{12} \pm 1.3 \times 10^9\ cm^{-2}$ and represent the position of the charge neutrality point (CNP) of tBLG that is related to disorder density. The distorted shape of the curve for hole doping (marked with blue parabola in Fig. **S4**(a)) is due to creation of depletion layer at negative backgate voltages. On (b) and (c) the frequency shift spectra for $\nu = 3/4$ and $4/4$ band filling are shown, respectively. The slight frequency jumps are associated to the large peaks in the dissipation spectra.

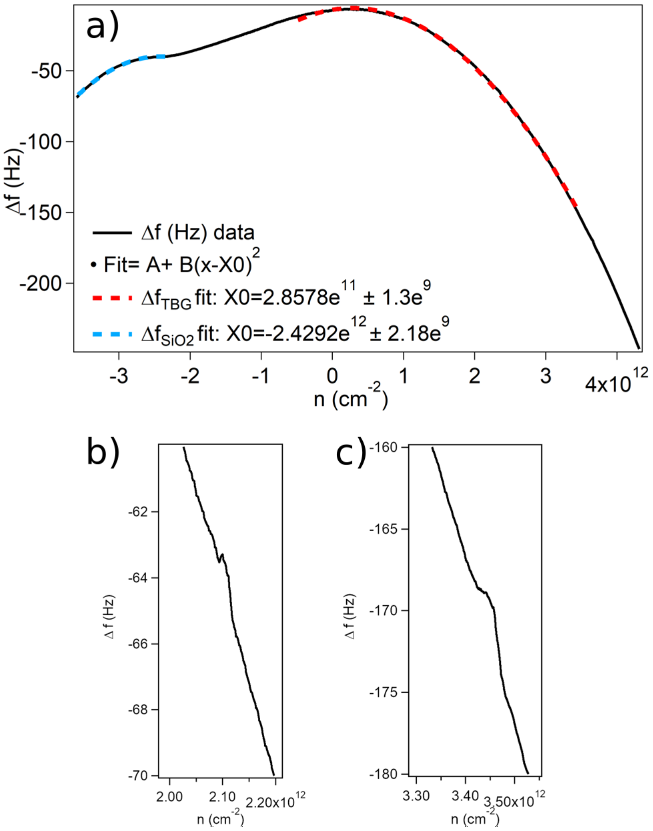

Figure S4. — $\Delta f(Hz)$ data versus doping and the spectrum was fit with two parabolas and the assymetric shape is due to creation of depletion region and is marked with blue parabola. X_0 refer to the parabolas maxima. (b) and (c) show the jumps in frequency shift for $\nu = 3/4$ and $4/4$, respectively.

SUPPLEMENTARY NOTE 4: LINE PROFILE AND TOPOGRAPHY

In order to determine the twist angle distribution we acquired 55 dissipation spectra along the line shown in Fig. **S5**. The pAFM topography image is shown in the center of Fig. **S5**. The raw data example spectra is shown on the right side of the Figure.

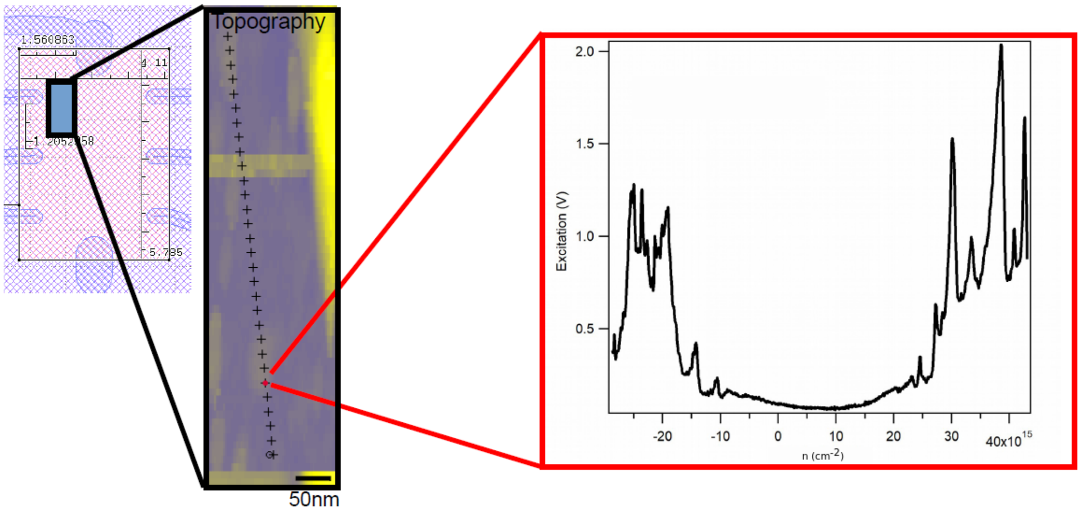

Figure S5. — On the left panel the schematics of tBLG device is shown. The rectangle marks the scanned area and the topography image is shown in the middle of the figure. 55 subsequent dissipation spectra were taken along the line profile shown in the topography image. An example of dissipation spectrum is shown on the right side of the figure.

SUPPLEMENTARY NOTE 5: EXCITATION AND FREQUENCY SHIFT CONSTANT HEIGHT IMAGES

Figure **S6** (a) and (b) show the dissipation images shown in Fig.2 of the main manuscript. (c) and (d) are the corresponding frequency shift data.

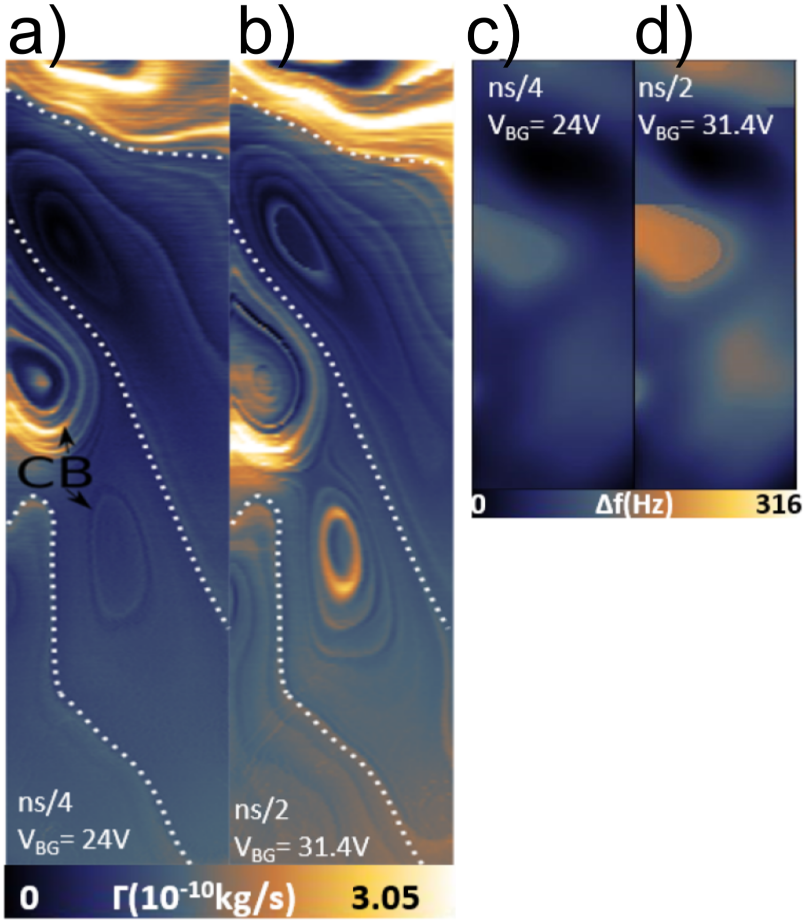

Figure S6. — Constant high p-AFM images taken at a d=150 nm distance. (a) is the dissipation constant height image over the sample surface for $n_s/4$ filling and taken at a V_{BG} = 24V. The white dashed lines are highlighting the different domains and the black arrows point to Coulomb rings. b) shows constant height dissipation image over the sample surface for $n_s/2$ filling taken at a V_{BG} = 31.4V. (c) and (d) are the corresponding frequency shift $\Delta f(Hz)$ constant height images taken for $n_s/4$ and $n_s/2$, respectively. The bright and dark regions visible in the frequency shift images are due to spacial variation of the attractive electrostatic force, which is due to different charge concentration.

SUPPLEMENTARY NOTE 6: MAGNETO-OSCILLATIONS

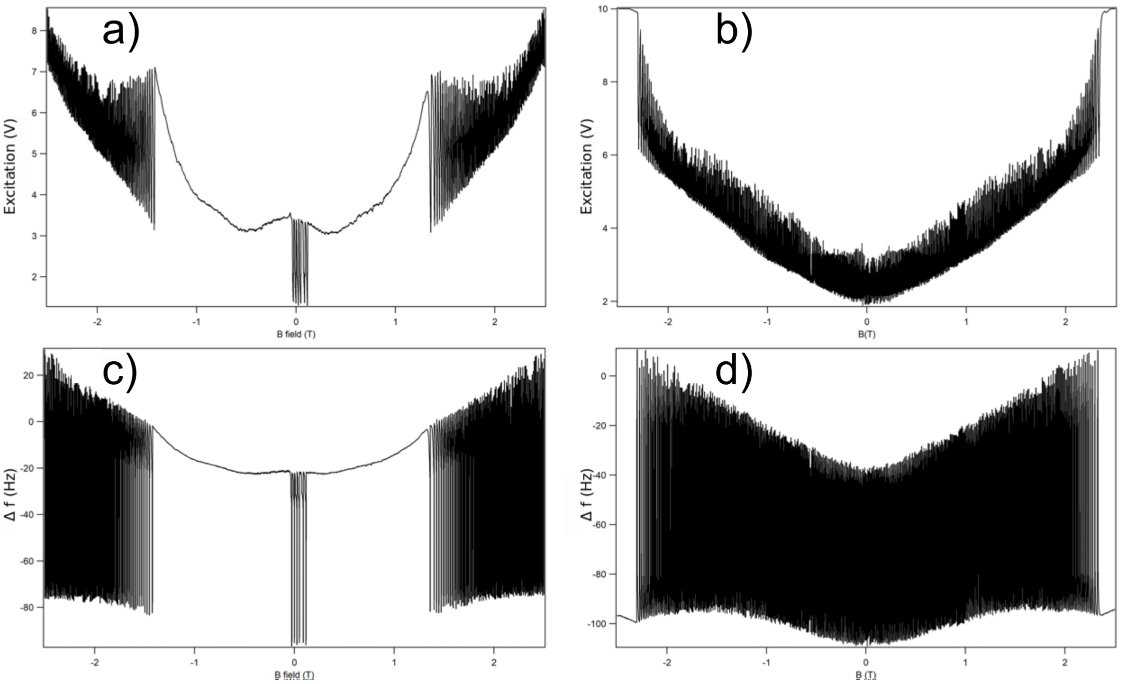

Figure S7. — Raw data magneto-oscillations visible in excitation signal (a,b) and frequency shift Δf signal (c,d). The data were acquired for fractional filling $\nu = 3/4$ (a,c) and for filling slightly larger, namely $3/4 < \nu < 4/4$ (b,d). The B-field was swept from -2.5T to 2.5 T.

SUPPLEMENTARY NOTE 7: ENERGY DISSIPATION VERSUS TIP-SAMPLE DISTANCE AND DOPING CONCENTRATION

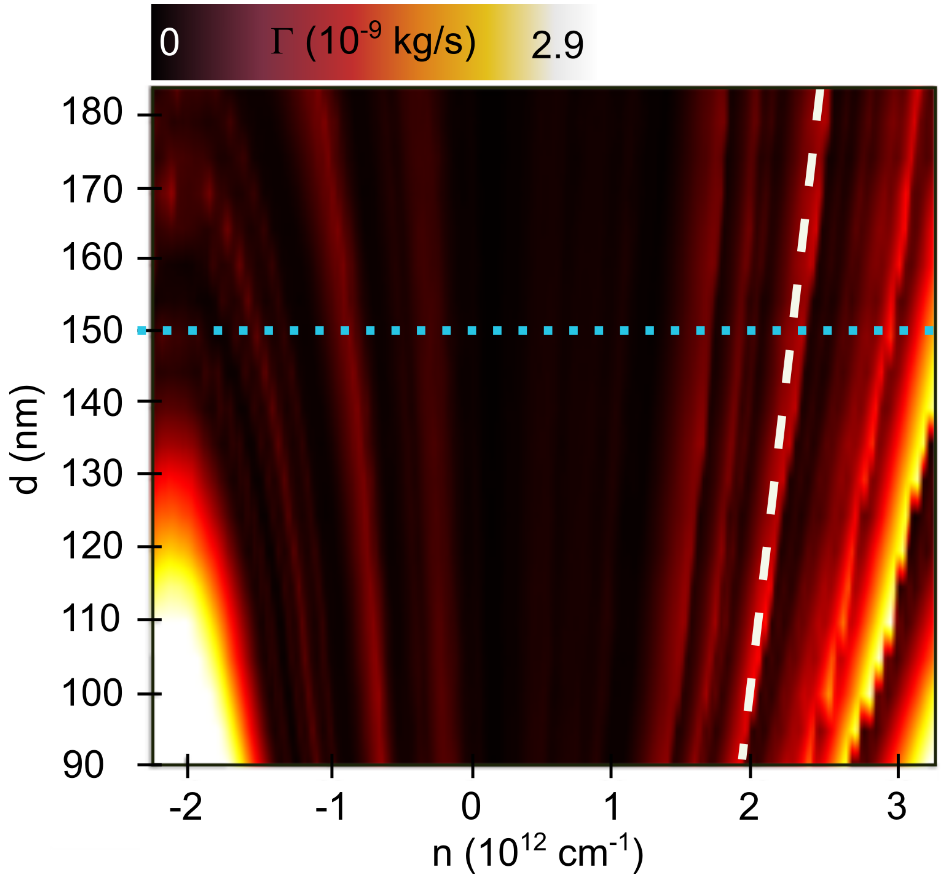

Figure S8. — The energy dissipation map versus doping n and tip-sample distance d. The dissipation peaks are visible as a bright lines at large tip-sample distance. The blue dashed line marks the distance $d = 150nm$ at which series of SIS was detected in dissipation. The white dashed line marks the change of the lever arm, which starts to deviate from linearity at very close tip-sample distances ($d < 100nm$).

SUPPLEMENTARY NOTE 8: DISPLACEMENT CURRENT, JOULE DISSIPATION AND FREQUENCY SHIFT

Dissipated power due to Joule looses is equal to:

$$P = I^2 \cdot R = V_B \cdot I \tag{S7}$$

where I, R, V_B stand for displacement current, resistance of the sample in the current path and bias voltage. When a voltage V_B is applied between tip and sample the displacement current induced by the oscillating tip is equal to:

$$i_d = \dot{q}(t) = \dot{C}\left(V_B - V_{CPD}\right) = \frac{\partial C}{\partial z}\frac{\partial z}{\partial t}\left(V_B - V_{CPD}\right) \tag{S8}$$

where C is tip-sample capacitance, C_{CPD} is the tip-sample contact potential difference equal to the difference of tip and sample workfunctions $\Delta\phi$. The tip oscillations are equal to: $z(t) = Asin(\omega t + \Phi)$ and then the dissipated power reads as follows:

$$P = R \cdot i_d^2 = R\left(\frac{\partial C}{\partial z}\right)^2 \left(V_B - \Delta\phi\right)^2 \left(\frac{\partial z}{\partial t}\right)^2 \tag{S9}$$

where $\gamma = R\left(\frac{\partial C}{\partial z}\right)^2 \left(V_B - \Delta\phi\right)^2$ is the effective damping coefficient proportional to the resistance of the sample in the current path. Next the dissipated power is:

$$P = R\left(\frac{\partial C}{\partial z}\right)^2 \left(V_B - \Delta\phi\right)^2 \left(-A\omega^2 sin(\omega t + \Phi)\right) \tag{S10}$$

and the dissipated power averaged over one period of oscillations is:

$$< P >= \frac{1}{2} R A^2 \omega^2 \left(V_B - \Delta\phi\right)^2 \left(\frac{\partial C}{\partial z}\right)^2 \tag{S11}$$

In case of capacitively coupled oscillating cantilever tip and the sample the energy of the system is given by $E = \frac{1}{2} C \left(V_B - \Delta\phi\right)^2$ and the force is $F = -gradE$. Thus:

$$F = -\frac{1}{2}\left(\frac{\partial C}{\partial z}\right)\left(V_B - \Delta\phi\right)^2 \tag{S12}$$

The oscillation frequency of the sensor is determined by the effective change of cantilever stiffness due to force interaction:

$$f = \frac{1}{2\pi}\sqrt{\frac{k + \frac{\partial F}{\partial z}}{m}} \tag{S13}$$

Taking into account, that frequency shift is defined as $\Delta f = f - f_0$, where f_0 is the free cantilever oscillation frequency, we can write:

$$\frac{\Delta f}{f_0} + 1 = \sqrt{1 + \frac{k_{ts}}{k}} \tag{S14}$$

where $k_{ts} = \frac{\partial F}{\partial z}$. Developing the right side of the equation into series and assuming weak force interaction compared to stiffness $k_{ts} << k$ the frequency shift is given by:

$$\Delta f = -\frac{f_0}{2k} k_{ts} = -\frac{f_0}{2k} \left(\frac{\partial F}{\partial z} \right) \tag{S15}$$

Finally, for capacitively coupled tip and sample:

$$\Delta f = \frac{f_0}{4k} \left(\frac{\partial^2 C}{\partial z^2} \right) (V_B - \Delta\phi)^2 \tag{S16}$$

SUPPLEMENTARY NOTE REFERENCE

---

* alexina.ollier@unibas.ch

† marcin.kisiel@unibas.ch

‡ xiaobolu@pku.edu.cn

§ urs.gysin@unibas.ch

¶ martino.poggio@unibas.ch

** dmitri.efetov@lmu.de

†† ernst.meyer@unibas.ch

[1] Gysin, U., Rast, S., Kisiel, M., Werle, C. & Meyer, E. Low temperature ultrahigh vacuum noncontact atomic force microscope in the pendulum geometry. *Rev. Sci. Instrum.* **82**, 023705 (2011).

[2] Kisiel, M. *et al.* Dissipation at large separations. In *Fundamentals of Friction and Wear on the Nanoscale*, 609–627 (Springer International Publishing, 2014).

[3] Kisiel, M., Samadashvili, M., Gysin, U. & Meyer, E. Non-contact friction. In *Noncontact Atomic Force Microscopy*, 93–110 (Springer International Publishing, 2015).

[4] Sze, S. M., Li, Y. & Ng, K. K. *Physics of Semiconductor Devices* (Wiley Sons, Limited, John, 2021).
